# Co-creating a patient and public involvement and engagement ‘how to’ guide for researchers

**DOI:** 10.1186/s40900-020-00208-3

**Published:** 2020-06-17

**Authors:** Raphaela E. Kaisler, Benjamin Missbach

**Affiliations:** Ludwig Boltzmann Gesellschaft (LBG), LBG Open Innovation in Science Center, Nussdorferstraße 64/2, 1090 Vienna, Austria

**Keywords:** Patient and public involvement, Co-creation, Stakeholder involvement, Multidisciplinary research

## Abstract

**Plain language summary:**

Research should benefit society at large. Involving citizens those who are affected by research may not only increase the quality, but can also push research towards generating greater societal benefits and relevant outcomes for citizens. Including citizens in research also has ethical implications, which necessitate structured guidance on ‘how to’ meaningfully involve them. In our project, we invited a multi-stakeholder group consisting of researchers from multiple disciplines, citizen scientists, youth and patient advocates to co-create a guide on ‘how to’ meaningfully involve citizens in research. In five consecutive workshops, we discussed how the characteristics of interactions between researchers and citizens (e.g., building trustful relationships and communication) and what a possible project steering structure enabling meaningful public involvement in research could look like. As a result of these workshops, the PPIE ‘How to’ Guide for Researchers was developed to support the implementation of ‘Patient and Public Involvement and Engagement’ (PPIE) activities and informed a PPIE Implementation Programme funding public involvement activities in Austria.

**Abstract:**

Involving citizens in research is not widely utilised across research disciplines and countries. It requires the readiness of researchers and their organisations as well as guides on ‘how to’ successfully involve citizens in a meaningful way. Including the patient and citizen voice in research activities has been most frequently demonstrated in health research, however, is implemented along various degrees of involvement – from passively receiving information about science to actively involving the citizens in steering projects and research activities. In this commentary, we aim to report a multi-stakeholder co-creation process developing ‘Patient and Public Involvement and Engagement’ (PPIE) activities across disciplines to provide guidance for researchers and the public. We use Ludwig Boltzmann Society’s (LBG) organisational framework as a case study, hence it consists of research institutes ranging from the life sciences to humanities and therefore represents a well-suited research environment for this endeavour. In a co-creation approach – to accomplish a shared understanding of public involvement in research among different stakeholders – a multi-stakeholder group comprising 11 researchers from natural sciences, life sciences, social sciences and humanities, and 13 citizens (such as patient advocates, young people and citizen scientists) were involved. In five consecutive workshops, we co-developed the nature of interactions between citizens and researchers, as well as governance structures enabling meaningful involvement in research. The workshops’ content was informed by an initial literature review. As a result of this process, the PPIE ‘How to’ Guide for Researchers was developed to support the implementation of involvement activities in their research projects according to the public involvement principles. These principles informed assessment criteria for the newly established PPIE Implementation Programme at LBG. It provides funding and support for public involvement activities in research to embed a sustainable and meaningful implementation of public involvement activities in Austria.

## Background

How can researchers meaningfully involve citizens in research projects across various disciplines? Working together in a way that values all contributions, and that builds and sustains mutually respectful and productive relationships challenges traditional ways of conducting research. However, a meaningful collaboration between researchers and citizens holds the potential to empower people and democratize knowledge on the one the one hand [1], but also to generate new forms of impact on the other hand [2, 3]. These new forms may include scientific as well as societal impact or benefit for society. This demands new ways of cooperating with citizens as stakeholders that have not been considered in research before and alters how research projects are set up and implemented pushing them towards a more collaborative effort to tackle societal challenges with research [4]. Research has shown that collaborations between citizens and researchers from multiple disciplines are needed to create societal impact [5]. Likewise, this discourse and the implementation of public involvement in research has led to a variety of effects for funders [6]. In particular, researchers often struggle to accommodate impact-oriented research as outlined in the new European Research Framework (Horizon Europe), which aims to fund research that is aligned with societal needs [7]. Researchers’ needs and methodological approaches depend upon and vary largely according to discipline traditions. Although it is challenging to develop public involvement principles that are valid for all disciplines and stakeholders, we think it is important to agree on core principles for implementing public involvement quality standards in research and enable bottom-up funding programmes open to all disciplines supporting the implementation of public involvement activities (in Austria). In this commentary, we aim to report on a multi-stakeholder co-creation process developing public involvement activities across disciplines to provide guidance for researchers and the public.

### What is public involvement in research

Involving citizens in co-creating research is currently discussed as one of the drivers for innovation within Europe, guiding mission-oriented research [4, 8]. Public involvement focuses on active and meaningful involvement of citizens in research processes and activities across the research cycle. According to the definition of the National Institute of Health Research [9] ‘user or public and patient involvement in research means doing research ‘with’ patients and the public so they are not just participants in the research. This requires users to have a say in the decisions made about research, so that the methods and outcomes are more appropriate to research participants and patients (p. 1).’

From a researcher perspective, introducing public involvement components into research projects may increase the overall empowerment of those who are affected by research, potentially fostering the democratisation of knowledge [10] and introduces a shift of power and ownership towards citizens. While knowledge is assumed to be widely distributed across society, the production of new knowledge through transdisciplinary projects has been pointed out since the early 90’s [11]. Researchers may involve citizens in different research activities and multiple phases in the research cycle.

Public involvement describes different ways of participation of citizen in research: ‘participation’ activities where citizens take part in research studies (e.g., clinical studies), ‘engagement’ activities where citizens perceive information and knowledge about research and dissemination (e.g., newsletter, social media), and ‘involvement’ activities that actively involve them in research activities as partners and in decision-making (e.g., representation in project steering boards). Several structured guidance for reporting involvement activities within research projects, for example the Guidance for Reporting Involvement of Patients and the Public (GRIPP) [12] and the revised version GRIPP2 [13], have been used to report involvement activities in a meaningful and comprehensible way.

A body of literature shows that citizen involvement not only leads to increased empowerment [14], but can also lead to better quality in research projects [15], which has been shown in health research [16]. For example, involving patients in the reviewing process in scientific journals like the British Medical Journal [17], the Research and Involvement and Engagement Journal [18], or in conducting systematic reviews via the Cochrane Crowd [19] aims to address patients’ needs and generate patient-relevant outcomes. In addition, Experience-Based Co-Design (EBCD) processes aim to improve the quality of healthcare by systematically applying a step-wise co-design process [20], mainly focused on involving patients and service providers. Likewise, the Priority Setting Partnerships (PSPs) collaborate with patients to conduct one and a half year prioritizing projects to establish top research priorities in various medical fields [21]. Both approaches mainly aim at providing methods and processes for the involvement of patients leading to health-related outcomes. While these efforts are highly relevant for health research, public involvement practices can be applied across various disciplines. However, there is a lack of practical guidance on how to best organise, assess and monitor citizens’ collaboration across disciplines and along different levels of involvement. To fill this gap, this commentary aims to report on co-creating a public involvement guide for researchers from various disciplines involving a large variety of different stakeholders along the process. We used the wording ‘Patient and Public Involvement and Engagement’ (PPIE) for describing the content of the guide, hence the concept of public involvement is not widely known and implemented in Austria. Therefore, we aim to introduce public engagement and involvement activities in the Austrian research landscape, thus serving as a basis for future public involvement funding programmes in Austria.

### Purpose of the PPIE Guide

Many public involvement guides are published and available to the scientific community [22–24], however, they mostly focus on engaging patients in one particular research field (e.g., health research) and do not capture strategies of involvement across disciplines. Therefore, we include more research and public perspectives in developing this PPIE Guide, which suits LBG’s goal of conducting research that benefits society in order to steer and generalise PPIE activities across disciplines. We emphasise the importance of co-creation – as a collective process developing and agreeing on content together with a group of stakeholders and experts– to establish a common understanding of public involvement among stakeholders in this process. Involving a broad variety of stakeholders ranging from young people, citizens, patients and researchers in a co-creative manner has shown to produce a different kind of output, also in the context of research [25].

The co-creation process aimed to, first, develop a PPIE ‘How to’ Guide that can be applied by researchers valuing all contributions – form research to public. Second, it aimed to raise awareness about public involvement activities across disciplines and therefore build a public involvement community of actors at LBG implementing public involvement activities in their research projects. Using this approach, we aimed to create a common understanding between researchers and citizens that serves as a basis for future public involvement activities within the LBG and beyond. Last, we aimed to build on the co-created public involvement guide, establishing a systematic PPIE Implementation Programme at LBG that funds public involvement activities in research in Austria. In this program, LBG supports researchers on an individual level facilitating implementation of such activities by offering consultation, training and peer support aiming to establish a public involvement community in Austria.

## Method

### A multi-stakeholder approach

To establish a systematic framework across disciplines and standards for meaningful involvement of citizens, we designed a series of five co-creation workshops comprising of different stakeholder groups: 1) researchers from natural sciences, social sciences and humanities, 2) patients representatives and patient advocates, 3) members of the public, especially, young people and citizen scientists (S1 Table). We searched for interested stakeholders within LBG institutes and our network – people that have been previously involved in other public involvement activities, projects and initiatives at the LBG – with experience of public involvement as participants or as citizen science investigators with basic knowledge on scientific processes.

In order to equally value all voices and needs from different stakeholder groups and to create a level playing field, citizens and researchers from all three disciplines shared leadership and project management tasks. The project team was comprised of a core team of two project managers and three researchers from LBG, patient advocates and four members of the public. Recruiting for the core team took place in the first and second co-creation workshops. Together the group co-created the content and activities for the co-creations workshops, participated in the co-creation workshops and collaboratively wrote the PPIE ‘How to’ Guide for Researchers. All other stakeholders constituted the steering committee that participated in the workshops, and gave feedback on the PPIE Guide’s during and after the co-writing process.

**Fig. 1:**
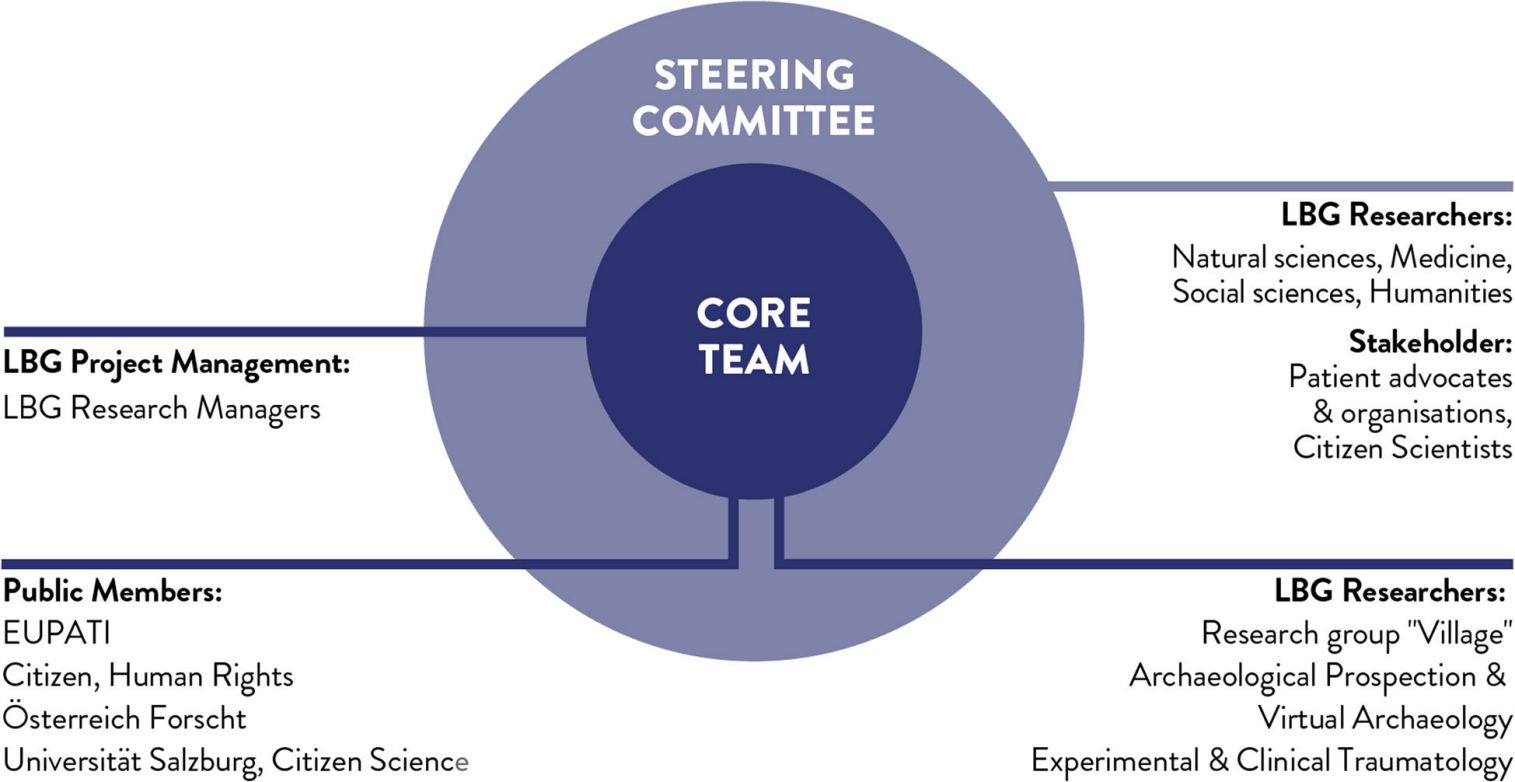
The multi-stakeholder approach. The LBG project management, core team, and steering committee co-led the project management

In total, 24 different stakeholders participated in the co-creation workshops. Thereof, 11 researchers from various disciplines and different level of involvement experience, such as natural and life science, humanities, artistic research and medicine, and 13 citizens representing citizen scientists, patient advocates, undergraduate students from different disciplines and high school students from 16 to 19 years age with basic scientific knowledge, formed the steering committee (Fig. 1). Knowledge on public involvement differed among the stakeholder groups, therefore, we decided to dedicate the first workshop to familiarising researchers with the public involvement concept and best practices examples from different disciplines. The stakeholders were informed about the literature in the field and the project prior to the workshop. In case of disagreement on specific issues, we continued discussing open question in the following workshop. In general, the stakeholder groups mutually agreed on the content created in the workshops, however, in some cases we collectively decided not adding conflicting content to the PPIE Guide as it is supposed to cover a broad range of disciplines.

### The co-creation process

**Fig. 2:**
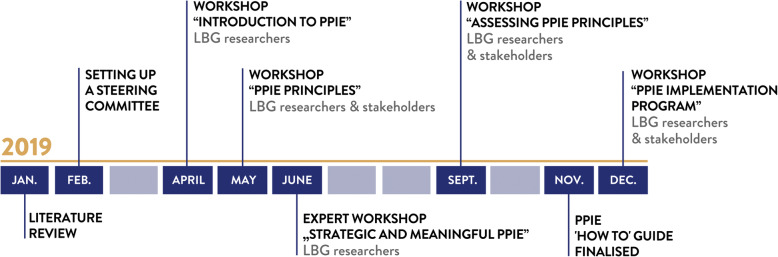
Overview and timeline of the co-creation process to develop a PPIE ‘How to’ Guide for Researchers

To systematically implement public involvement activities at LBG, we took a multi-stakeholder approach and began a co-creation process in the beginning of 2019. The co-creation process comprised of the following steps (Fig. 2): First, the authors conducted a brief literature review on public involvement practices. This literature review helped us to design the outline of the co-creation process. Second, we contacted all LBG institutes to establish interest in them becoming part of the public involvement steering committee co-creating a PPIE ‘How to’ Guide as well as learn from existing public involvement activities and initiatives carried out within the LBG research institutes. Third, we designed a series of five workshops addressing different topics – from co-creating public involvement principles to monitoring them and building a foundation for systematically implementing public involvement at the LBG and beyond. Note that the outcome of the first co-creation workshop informed the content of the second and so forth, making this process tailored to the stakeholder groups’ needs and interests (Table 1). In general, the workshops were designed along this structure: 1) welcome and orientation of participants, information and input about the project and progress, 2) breakout sessions in small groups to co-create content according to guiding questions, 3) reflection and feedback on the co-created output from breakout sessions in the plenum, and 4) brief outlook and next steps. We facilitated the discussions in small groups of 4–6 people and documented the key discussion points on flip charts. Afterwards, we presented the results from all group discussions to all stakeholders groups and collected feedback and open questions. A detailed methodological description is summarised in the supplementary material (S2 Table).

**Table 1:** Overview of the co-creation workshops, target groups, aims and outcomes

Workshop	Target Group	Aims	Outcome
**1**	LBG researchers	Introduction of public involvement concepts, benefits and challenges	• Researchers expectations in the co-creation process • Assessment of needs to implement PPIE in their research projects
**2**	LBG researchers & stakeholders	Co-create principles of meaningful public involvement	• Definition of public involvement principles on meaningful interactions between researches and citizens • Identification of public involvement governance structures in research projects • Identification of organisational support structures
**3**	LBG researchers	Expert talk: Public involvement on an organisational and strategic level	• Awareness raising and onboarding of senior researchers • Highlighting benefits and challenges of public involvement
**4**	LBG researchers & stakeholders	Co-creating monitoring criteria of good public involvement practice	• Refinement of public involvement principles • Monitoring of the quality of public involvement activities
**5**	LBG researchers & stakeholders	Concept of a PPIE Implementation Programme	• Funding model for public involvement activities • Support structures complementing funding • Evaluation of public involvement activities

In the first workshop, we introduced the concept, benefits and challenges of public involvement to the researchers from different disciplines mentioned above. We decided to first familiarise and align researchers’ knowledge with the concept before introducing other stakeholder groups (citizens and patients) in the workshops. This on boarding process of researchers aimed to build a public involvement workforce at LBG. Good practice examples of involvement, for example form orthopaedic traumatology, mental health research, and cancer research, gave an overview of how to involve citizens in different stages of the research cycle, such as in priority setting, co-creation of research activities and dissemination. In this workshop, we focused on the expectations and needs from the researcher perspective regarding public involvement that gave us a first impression and content for the next co-created workshop with other stakeholders.

In the second workshop, we organised a meeting bringing together researchers and stakeholders such as patient advocates, citizen and youth interested in research. The second workshop aimed to co-create principles of meaningful involvement in research and project steering structures when applying public involvement activities in research projects. We focused on an individual, project, and organisational level addressing trustful and transparent interactions between citizens and researchers, making governance structures inclusive and identifying organisational support structures, necessary to implement meaningful involvement activities in research.

In the third workshop, we invited the internationally recognised and highly experienced youth involvement expert, Ian Manion (Canada) covering the big-picture of public involvement importance on an organisational and strategic level. Senior researchers of the LBG institutes attended the event.

After the third workshop, the core team structured and summarised the content created in the previous workshops and provided a structure for the PPIE Guide based on the outcomes of the workshop online using a collaborative tool (Google Docs). Afterwards we invited all stakeholders to comment on the structure and content of the PPIE Guide online. To discuss the current content of the PPIE Guide with all stakeholders, we dedicated a session in workshop 4 and 5 to give feedback.

In the fourth workshop, we co-created monitoring criteria of good public involvement practice together with representatives from all stakeholder groups. First, we refined the co-created public involvement principles and in a second step discussed how and who could monitor the quality of involvement activities. To openly access and distribute the PPIE Guide in the scientific and other communities, the ‘How to’ Guide for Researchers is published under CC-BY 4.0 licence ‘free to share and adapt’.

In fifth and final workshop, we discussed potential funding models with all stakeholder groups based on the PPIE ‘How to’ Guide. The discussion covered three topics co-creating action plans to establish a public involvement focus at LBG: funding structures to implement public involvement activates, support structures to facilitate implementation, and evaluation of public involvement activities. The output led a nationwide PPIE Implementation Programme funding and supporting public involvement activities in research launched in autumn 2020.

## Results

In this project, the public involvement principles developed in the co-creation workshops informed the structure and content of the PPIE ‘How to’ Guide for Researchers. The public involvement principles address the following issues: the interactions between researchers and citizens, and the governance structures enabling meaningful involvement in research. It describes the necessary considerations to actively involve citizens on an individual, a project, and an organisational level. A detailed description is provided in the PPIE ‘How to’ Guide for Researchers and is freely available via zenodo [26] via the link: https://zenodo.org/record/3515811.

### Interactions between citizens and researchers

Interactions between citizens and researchers are key for this approach to meaningfully involve them in specific research activities. We refer to e.g. patients as people with a mental or physical illness or people with lived-experience in a certain field, e.g. mental health, and the public as people with a general interest in research or those people affected by research. Following public involvement principles and general considerations have been identified involving citizens in research: researchers offering involvement activities for citizens at different steps in the research process (once or multiple involvement is possible) may identify gaps of knowledge and experience needed in the project. It allows for checking the current research activity with respect to societal relevance. Involving citizens from the beginning, e.g., in the ideation phase, grant and funding application writing, and before the project starts helps to design a user-centred approach and inclusive research activities [27, 28]. To support the collaboration between these parties, researchers should provide mutual learning activities and career development opportunities, such as co-led talks and conferences, as well as visit events and workshops. Furthermore, citizens’ contributions to the project need to be visible and (monetarily and non-monetarily) honoured, i.e., honorariums, and authorship for citizens or consortium on publications. These contributions should be published under a creative common licence. The support of citizens’ contributions should take their availability regarding time and place into account.

On an individual level, researchers should consider fostering open and honest communication (creating a level playing field) that allows the building of trust between researchers and citizens. The establishment of ‘flat hierarchies’ helps in the comprehension and value of each other’s expertise by providing clear expectations (also in regard to reimbursement of time). Hierarchical structures are not solely driven by expertise, skills or experience. They should be developed with respect to shared-decision making and equal rights regarding the project outcomes and processes. Researchers should focus on transparent communication addressing potential conflicts of interest and avoiding the use of academic/research/medical jargon to describe the research project and results (especially when disseminated to the wider public). Continuous communication throughout and after the project supports collaboration. Researchers should provide job ‘clarification of activities or tasks they will be involved with’ to avoid miscommunication and disappointment in expected tasks and activities, and allow adaption of these tasks and roles during the collaboration. To demonstrate the value of citizens’ input, researcher should always inform citizens about adoption of research activities based on their feedback and communicate ways in which feedback has been incorporated into the research process.

### Recruitment strategies for involvement activities

Recruitment strategies should consider defining a target group (potential people to involve), relevant demographics, such as age, patient within the same field or disease, geography, previous experiences, diversity, and equality. In order to gather different perspectives in the project, researchers should recruit a suitable number of citizens (broader network) and not just a single individual for a task or in the project. Tactics and approaches for recruitment may vary according to the group of patients and may be influenced for example by their stage of recovery and health status. In order to find suitable citizens for a research project, researchers should think about the individual and existing skills matrix that is needed and invest in support activities and orientation processes. Citizens might need specific training and skills for fulfilling tasks in the project, therefore, the research team should co-create the format, training agenda and content needed together with citizen representatives. In general, training should be tailor-made, modular, needs oriented and co-creative. Researchers should consider co-leading the training with an experienced patient or a member of the public interested in research. Patient or public ‘champions’ may also support in recruiting and could act as an entry point for new members. The moderation of the training might be outsourced depending on the researchers’ facilitation skills and include a person with lived-experience or from the public. The training might be provided at the beginning or during various stages throughout the project.

### Governance structure of PPIE projects

On a project level, to enable citizens to get actively involved in research, the following governance structure should be established in a project. A Project Steering Board should include at least two citizens (e.g., patients with lived-experience or members of the public). The board should meet regularly (recommended twice a year) and advise the research team on the planned project activities and collaboration.

A Study Advisory Group consisting of 3–6 citizens (e.g., people with lived-experience on a specific topic required in the project). They consult the project team on a regular basis (i.e., once a month, or as appropriate and feasible for the individuals and the research project). The Study Advisory Group should be established before the project starts. Furthermore, working for instance with patients or a person with lived-experience requires an appropriate safety plan (depending on the topic and research area), for example, for physical or mental wellbeing; a clinician should be on call in case of emergency or be present at big events. External supervision should be provided for citizens on demand and on a regular basis to reflect on their roles and content (e.g., every 8 weeks).

Organisations considering implementing public involvement as an integral part of their research should consider the following institutionalised structures to enable meaningful involvement activities in research projects: a coordination of involvement activities (PPIE Officer) and contact point for complaints and concerns from patients (Patient Ombudsman). The PPIE Officer coordinates public involvement activities at an organisational level and has an oversight on all public involvement activities in the organisation. The PPIE Officer acts as a consultant and advisor and may be approached by researchers and interested citizens. The Patient Ombudsman is a neutral contact person that can be addressed in case of complaints and concerns. He/she is an independent external person. He/she investigates complaints from individuals and organisations about maladministration by the research organisation. Maladministration occurs if an institution or researcher fails to act in accordance with the Declaration of Helsinki or the principles of public involvement, or violates human rights. Maladministration can include administrative irregularities, unfairness, discrimination or the abuse of power, for example in the managing of public involvement funds, procurement or recruitment policies.

Support structures provided by organisations, such as regular network meetings and learning events, foster mutual exchange while carrying out public involvement activities. For example, offering a family-friendly work environment by setting up or reimbursement of childcare costs might support citizens to take part in research activities. Additionally, a public platform introducing public involvement projects and activities may increase the visibility of projects and opportunities to become involved in research. For instance, presenting the involved project participants, the project process by displaying the most important steps, the methods used and an online space for exchange and learning. The platform could inform and connect different stakeholders in terms of available opportunities in research projects and virtual matchmaking events with researchers and research projects.

### Monitoring PPIE activities

Successful public involvement activities in research need a structured means of monitoring the quality and implementation of public involvement activities. A representative of the Study Advisory Group (citizens), PPIE Officer and the Principal Investigator of the research project together monitor the implementation of public involvement activities in the research project. In regular meetings (e.g., suggestion 2-4x per year and on demand), the team evaluates past and current public involvement activities and discuss further steps for implementation and improvement. They may consult with the Patient Ombudsman discussing conflicts between the parties and/or individual complaints. The team documents their results and gives recommendations for further the implementation of future public involvement activities. A comprehensive tool for self-assessment of self-involvement activities on a project and organisational level, and a checklist to prepare involvement activities before, during and after the project may be used for monitoring purposes and can be found in the PPIE ‚How to’ Guide for Researchers.

### Concept of a PPIE Implementation Programme

The PPIE Implementation Programme aims to support implementation of public involvement activities with citizens in the area of ‘active involvement’ across different phases of the research cycle (from setting the agenda to interpreting data) and its governance. It supports public involvement activities with up to EUR 60.000 over a project period of max. 12 months implemented at Austrian research organisations and universities. An independent panel of experts, consisting of representatives from two scientific experts in the field public involvement, a citizen and patient in the field of health, and two young people (16–25 years) with basic scientific knowledge, assesses the applications based on the quality of involvement, societal impact, implementation plan, and feasibility within this time frame given. The evaluation of public involvement activities includes views from all stakeholders that participated in the activities (researchers and citizens) and addresses the following dimensions: quality of involvement, learnings from activities, future and sustainability of activities, scientific and societal impact of activities on individual and organisational level, implementation of activities, and satisfaction with the activities. In addition, and at the core of the programme is the aim to builds the institutionalised support at LBG. This support will take form of offering individual consultation for researchers and citizens, training opportunities, such as webinars and co-creation workshops with different stakeholder groups, as well as learning opportunities through a peer network to establish a public involvement community and embed public involvement in the Austrian research landscape.

## Discussion

This project aimed to co-create a shared understanding of public involvement activities across disciplines and establish public involvement at the LBG as an integral part of research projects. A group of stakeholders including researchers from various disciplines, patient advocates, citizen scientists and young people co-created principles for meaningful involvement of citizens in a series of five workshops. As an outcome, the PPIE ‘How to’ Guide for Researchers was co-created that serves as a public involvement framework at LBG and laid the foundation for a PPIE Implementation Programme funding and supporting public involvement activities in Austria.

### Learnings

Challenges in this project addressed the engagement of multidisciplinary researchers in public involvement activities that focus on the level of meaningful involvement of citizens. Due to the lack of expertise, good practice examples, as well as lack of organisational support structure in the Austrian research landscape, the concept of public involvement is currently not wide spread.

We used the co-creation process to introduce researchers to a new topic – public involvement in research – and acknowledged their different views form various disciplines in the workshops by matching multiple stakeholders from the public and researchers. It was important to first familiarise the researchers with good practice examples of public involvement from UK [22, 23] and Canada [24] before bringing other public stakeholders into the process. As expected, researchers in the field of natural sciences focusing on basic research had more difficulties in identifying potential public involvement activities in their research projects compared to researchers from the social sciences and humanities. In general, public involvement activities can be applied in all steps of the research cycle, however, for some parts of the steps it might be more realistic for natural scientists to involve citizens, such as priority setting, project steering and dissemination of results. Another example, supporting the creation of a shared vision was the shared leadership approach in this project. The project team was established in the first and second workshop including patient advocates, members of the public and researchers in the project steering. The intended distribution of power among the core team resulted in equal contributions of stakeholders and empowered citizens valuing others perspectives and having decision rights. As a secondary outcome, the empowerment process led to a newly established public involvement community and increased researchers’ interest in implementing public involvement in their research.

The co-creation process helped researchers raise awareness about benefits and challenges of implementing public involvement in their work and supported a common understanding. It allowed them to express their expectations for individual projects, their research in general and future opportunities at the LBG. For example, researchers expected innovative translational collaboration with other disciplines as a result of this co-creation process, as well as an increase in the visibility of their work when working with citizens. They emphasised that additional funding opportunities for public involvement activities should be provided to implement involvement activities in their work. It became clear from the beginning that support structures in the organisation and a wider framework that includes funding and facilitation of public involvement activities are needed in future to implement public involvement in research projects. This multi-stakeholder approach was the first co-created project with citizens in terms of project design at the LBG. From our perspective, this had substantial spill over effects for other projects planned within LBG, such as creating a funding model for future PPIE activities. For example, as an outcome of the fifth workshop, different stakeholder group addressed the key features and assessment criteria for a future public involvement program that systematically funds and supports researchers implementing public involvement activities in research. Building on these outcomes, LBG prioritised public involvement as one focus at the LBG Open Innovation in Science Center (https://ois.lbg.ac.at) and set up a PPIE Implementation Programme facilitating public involvement activities in research in Austria. The PPIE Implementation Programme and first national PPIE pilot call opening in September 2020 (https://ppie.lbg.ac.at). This co-creation process, which led to a funding programme, may act as a role model for future public involvement funding programmes including researchers’ and citizens’ needs and idea.

### Limitations

Even though the outcomes of this process resulted in important learnings, they come along with some limitations. First, we selected stakeholders based on their interest and experience with previous public involvement activities. Some stakeholders were more experienced than others, which resulted in a need for us to align the knowledge in the room about public involvement in the first workshop. Second, we did manage to reach a range of disciplines from the social sciences to natural science, however we did not reach all disciplines. Third, we did not explicitly co-create the assessment of quality criteria for future public involvement activities for the PPIE Guide, and the co-creation process itself. However, the dimensions of evaluating public involvement activities were discussed and co-created workshop, after the PPIE Guide was completed. The outcome of this workshop resulted in a concept of a PPIE Implementation Programme including an evaluation questionnaire for researchers and stakeholders (https://ppie.lbg.ac.at).

In fact, the co-created guide is helpful in order to establish monitoring criteria for public involvement activities, however, at this point does not include the necessary information on assessment tools or indicators. Third, we planned to insert good practice examples from our LBG research institutes in different steps of the research cycle. During the process, we retracted this idea because we wanted to collect examples from all disciplines involved in the project, which are currently planning their activities. We plan to update the PPIE Guide on a regular basis, once public involvement projects will be developed accordingly, we will report good practice examples. Fourth, the motivation to participate varied depending on the stakeholder groups. No explicit incentives were given to participate in the series of workshop (other than traveling costs for those not located in Vienna). We found that involving for instance patients directly was challenging due to the generic and mostly abstract challenge we were facing. To tackle this issue, we drew on already existing networks covering patient organisations and patient advocates. Furthermore, we could not reach all LBG research institutes, even though researchers from all institutes with different levels of seniority were invited to participate. Despite this limitation, we could cover a wide range of disciplines mapping very well on the various disciplines represented within LBG.

## Conclusion

The PPIE ‘How to’ Guide supports researchers on implementing involvement activities in their research projects. More importantly, the multi-stakeholder and co-creative approach helps building a research community that values citizens’ contributions and identifies support structures needed to sustainably implement public involvement in their work. As on outcome of this co-creation process, the PPIE Implementation Programme funding and facilitating public involvement activities was established. Such systematically developed bottom-up processes involving different stakeholders and disciplines are key to raising awareness, building a critical mass and implementing change on an individual and an organisational level.

## Supplementary information

**Additional file 1: Table S1.** Overview of different stakeholder groups that participated in the co-creation workshops.

**Additional file 2: Table S2.** Overview of the co-creation, one-day workshops’ design: participants, sessions, methods, and documentation.

## Data Availability

The PPIE ‘How to’ Guide for Researchers is freely available at our website ois.lbg.ac.at and at the European repository zenodo via the link: 10.5281/zenodo.3515811 [26].

## Abbreviations

LBG: Ludwig Boltzmann Society (in German: Ludwig Boltzmann Gesellschaft)
PPIE: Patient and Public Involvement and Engagement
