# Supplementary material for: Co-creating a patient and public involvement and engagement ‘how to’ guide for researchers
Source: Res Involv Engagem. 2020 Jun 17;6:32. doi: 10.1186/s40900-020-00208-3 (PMC7301967; doi:10.1186/s40900-020-00208-3)
Supplement: Supplementary file 1 — Additional file 1: Table S1. Overview of different stakeholder groups that participated in the co-creation workshops. [file 40900_2020_208_MOESM1_ESM.docx]

**S1 Table. Overview of different stakeholder groups that participated in the co-creation workshops.**

|  |  | **Workshop** | | | | | |
| --- | --- | --- | --- | --- | --- | --- | --- |
| **Stakeholder groups** |  | 1 | 2 | 3 | 4 | 5 | |
| Young people (16-19 years) |  | 0 | 1* | 0 | 5 | 0* | |
| PhD-Students |  | 4 | 2 | 2 | 3 | 1 | |
| Senior researchers |  | 7 | 5 | 9 | 4 | 2 | |
| Citizens |  | 0 | 4 | 0 | 8 | 4 | |
| Patient advocates |  | 0 | 4 | 0 | 2 | 2 | |
| Total number of participants |  | 11 | 16 | 11 | 22 | 9 | |
| **Represented research disciplines** |  |  |  |  |  |  | |
| *Natural sciences*  (Oncology, pulmonology, genetics, clinical traumatology) |  | 5 | 3 | 7 | 4 | 2 | |
| *Social sciences*  (Health technology assessment, mental health, sociology) |  | 3 | 2 | 2 | 2 | 1 | |
| *Humanities*  (History, human rights, new Latin studies, archeology) |  | 3 | 2 | 2 | 1 | 0* | |
| *Note.* Figures indicate numbers of workshop participants. *Two people confirmed, but did not participate in the workshop. | | | | | | |  |
