# Supplementary material for: Co-creating a patient and public involvement and engagement ‘how to’ guide for researchers
Source: Res Involv Engagem. 2020 Jun 17;6:32. doi: 10.1186/s40900-020-00208-3 (PMC7301967; doi:10.1186/s40900-020-00208-3)
Supplement: Supplementary file 2 — Additional file 2: Table S2. Overview of the co-creation, one-day workshops’ design: participants, sessions, methods, and documentation. [file 40900_2020_208_MOESM2_ESM.docx]

**S2 Table. Overview of the co-creation, one-day workshops’ design: participants, sessions, methods, and documentation.**

| **WS** | **Participants** | **Workshop sessions** | **Method** | **Documentation** |
| --- | --- | --- | --- | --- |
| 1 | LBG Researchers | a. Welcome & orientation of participants:   - Introduction of person and organisation - Interest in research area | Brief introduction | Photo protocol |
|  |  | b. Short introduction to the PPIE project, Q&A | Power Point presentation & facilitated discussion | Slides in protocol |
|  |  | c. Breakout session: co-creation of three topics   - What are researchers’ expectations and needs regarding public involvement? - What is your personal and organisational benefits implementing public involvement in research? - What kind of resources do you need to implement public involvement in research? My contribution to the project? | Open Space method:   - Hosts stay at stations - Participants rotate between stations - Host facilitates and documents output of small group discussions | Flip chart protocol |
|  |  | d. Presentation of small group discussions to all stakeholders | Facilitated discussion in plenum |  |
|  |  | e. Organisational issues:   - Recruiting of participants to core team - Tasks of project management team - Communication structure in the PPIE project | Facilitated discussion in plenum | Flip chart protocol |
|  |  | f. Outlook and closing |  |  |
| 2 | LBG Researchers & stakeholders | a. Welcome & orientation of participants:   - Introduction of person and organisation - Interest in research area | Brief introduction | Photo protocol |
|  |  | b. Short introduction to the PPIE project, Q&A | Power Point presentation & facilitated discussion | Slides in protocol |
|  |  | c. Breakout session: co-creation of three topics   - How does the interaction between researchers and citizens/patients need to be structured, so that I can actively participate in research activities? - What quality criteria need to be fulfilled, so that I can actively participate in research activities over a longer period? - Which resources and conditions have to be provided, so that I can actively take part in research activities? | Open Space method | Flip chart protocol |
|  |  | d. Presentation of small group discussions to all stakeholders | Flip chart presentation & discussion |  |
|  |  | f. Organisational issues:   - Recruiting of participants to core team - Tasks of project management team - Communication structure in the PPIE project | Facilitated discussion in plenum | Flip chart protocol |
|  |  | g. Outlook and closing |  |  |
| 3 | LBG Researchers | a. Welcome & orientation of participants   - Introduction of person and organisation - Expectations for the workshop | Brief introduction | Photo protocol |
|  |  | b. Presentation of ‘strengthening research through meaningful  public and patient involvement in research’   - Q&A - Self-assessment of public involvement activities - Discussion | Power Point presentation  & facilitated discussion in plenum | Slides in protocol |
|  |  | c. Debrief and closing |  |  |
| *PPIE Guide* | *Core team* | *Co-write the PPIE ‘How to’ Guide for Researchers:*   - *Structure topics from workshops 1-3* - *Add outcomes from workshops* | *Online discussion with core team & collaborative writing* | *Google Docs* |
| 4 | LBG Researchers & stakeholders | a. Welcome & orientation of participants   - What does public involvement mean for me? - What is interesting about this project? - What is my research interest? | ‘Speed dating’ activity:   - Tandems - 2 minutes discussions - Switch to next participant |  |
|  |  | b. Feedback to PPIE ‘How to’ Guide for Researchers | Facilitated discussion in plenum | Flip chart protocol |
|  |  | c. Breakout session: co-creation of three topics   - Assessment of interactions between researcher and citizens - Monitoring structure for PPIE activities - On boarding and training concepts for researchers & citizens | Open Space method | Flip chart protocol |
|  |  | d. Presentation of group discussions to all stakeholders | Flip chart presentation & discussion |  |
|  |  | e. Outlook and closing |  |  |
| *PPIE Guide* | *Core team* | *Continue co-writing the PPIE ‘How to’ Guide for Researchers: Add outcomes from workshop 4* | *Collaborative writing* | *Google Docs* |
| 5 | LBG Researchers & stakeholders | a. Welcome & orientation of participants   - My research interest/area - My organisational role - My satisfaction with the PPIE project | Brief introduction | Photo protocol |
|  |  | b. Short presentation of public involvement funding models | Power Point presentation & discussion | Flip chart protocol |
|  |  | c. Feedback to PPIE ‘How to’ Guide for Researchers | Facilitated discussion in plenum | Flip chart protocol |
|  |  | d. Breakout session: co-creation of three topics   - Public involvement funding models - Institutionalised support of public involvement activities - Monitoring of and learning from public involvement activities | Open Space method | Flip chart protocol |
|  |  | e. Presentation of group discussions to all stakeholders | Flip chart presentation & discussion |  |
|  |  | f. Outlook and closing |  |  |

*Note.* WS = Workshop.
